# A decision tree-based algorithm for structured risk stratification of rare rheumatic diseases in a tertiary referral setting

**DOI:** 10.3389/fmed.2026.1734483

**Published:** 2026-07-02

**Authors:** Christine Babka, Markus Storck, Torsten Witte, Vega Gödecke

**Affiliations:** 1 Medizinische Hochschule Hannover (MHH), Zentrum für seltene Erkrankungen, Hannover, Germany; 2 Ostfalia Hochschule für Angewandte Wissenschaften – Hochschule Braunschweig/Wolfenbüttel, Fakultät Handel und soziale Arbeit, Suderburg, Germany; 3 Medizinische Hochschule Hannover (MHH), Klinik für Rheumatologie und Immunologie, Hannover, Germany; 4 Medizinische Hochschule Hannover (MHH), Klinik für Nieren- und Hochdruckerkrankungen, Hannover, Germany

**Keywords:** center for rare diseases, CHAID decision tree, rare rheumatic diseases, rheumatic pain, tree-based algorithm

## Abstract

**Background:**

Rare inflammatory rheumatic diseases are often characterized by heterogeneous, multisystemic symptom patterns, complicating early diagnostic differentiation. This study aimed to model a structured, symptom- and laboratory-based decision approach for risk stratification of rheumatologic diagnoses in patients referred to a tertiary Center for Rare Diseases (CRD) with unclear systemic complaints.

**Methods:**

We conducted a retrospective cross-sectional analysis of 173 patients evaluated at a CRD. Patients were classified into rheumatologic (RHEUMA) and non-rheumatologic (OTHER) diagnostic groups based on final diagnostic outcomes. A standardized questionnaire capturing 52 symptoms was aggregated into domain-specific and composite scores. Laboratory data were summarized into predefined indices. Group differences were analyzed descriptively using inferential statistics. A decision tree model based on Chi-Square Automatic Interaction Detection (CHAID) was constructed to support structured risk stratification.

**Results:**

A confirmed rheumatologic diagnosis was established in 52.0% of patients. Symptom and comorbidity patterns were broadly similar across diagnostic groups, with fatigue and generalized pain being the most prevalent complaints. The RHEUMA group showed significantly higher scores in selected symptom domains and in an immunoserological laboratory index (all *p* < 0.05). The CHAID-based decision model, integrating symptom scores, laboratory markers, and autoimmune history, showed an apparent classification of 81.5% (AUC = 0.893), compared to 76.3% (AUC = 0.823) for logistic regression.

**Conclusion:**

The proposed decision tree model provides a transparent framework for structured risk stratification in a highly preselected tertiary referral population. Given the monocentric, retrospective, and exploratory study design, the findings should be interpreted as hypothesis-generating. External validation in independent cohorts is required to assess model generalizability, calibration, and robustness before clinical implementation can be considered. Within these constraints, the approach illustrates the potential of transparent, rule-based symptom aggregation to support structured clinical reasoning and prioritization in complex multisystem presentations.

## 1 Introduction

Rare and diagnostically unclear diseases represent a persistent challenge in modern healthcare. Their low prevalence, combined with heterogeneous, non-specific, and often multisystemic symptom presentations, complicates timely diagnosis. While clear clinical symptoms facilitate rapid classification (1), non-specific symptoms affecting multiple organ systems make targeted clarification considerably more difficult (2–4). Structured care approaches in specialized centers have therefore been proposed to address diagnostic complexity in rare diseases (5, 6).

As a result, diagnostic processes are frequently prolonged, sometimes with substantial consequences for patients’ quality of life and long-term prognosis (7–12). The time to diagnosis for rare or unclear diseases ranges from a median of 4.5 to 8.2 years and often significantly exceeds this period (8, 9).

This challenge is particularly evident in the context of rare inflammatory rheumatic diseases (IRDs). These conditions are frequently characterized by systemic disease activity and involvement of multiple organ systems, often without a clear organ- or tissue-specific predilection (2, 12, 13). Their clinical heterogeneity substantially complicates differential diagnosis, as individual symptoms and organ-specific findings are often insufficient to distinguish between rheumatologic and non-rheumatologic conditions, particularly in patients presenting with heterogeneous and overlapping symptom constellations.

Delayed diagnosis and inadequate or delayed treatment have been shown to be associated with unfavorable outcomes, particularly in IRD (8, 14–16).

Against this background, there is a need for structured approaches that integrate clinical information, including patient-reported symptoms and laboratory findings, into a coherent framework. In complex multisystem presentations, individual symptoms and isolated findings often provide limited diagnostic discrimination, particularly in the presence of overlapping and non-specific symptom patterns. As a result, diagnostic differentiation frequently depends on the integration and contextual interpretation of multiple features rather than on single indicators. In highly preselected tertiary care settings, where patients typically present with prolonged diagnostic uncertainty and extensive prior evaluations, structured and transparent approaches may support the organization of clinical information and facilitate risk-oriented prioritization of diagnostic considerations.

Given this context, the present study sought to operationalize such a structured approach by developing and examining a symptom- and laboratory-based framework for risk stratification of rheumatologic diagnoses in patients referred to a Center for Rare Diseases (CRD) with unclear, multisystemic complaints. By aggregating patient-reported symptoms into clinically informed domain-specific and composite scores and integrating selected laboratory parameters, we aimed to identify recurring patterns that support differentiation between rheumatologic and non-rheumatologic conditions within a highly preselected tertiary care population.

A central component of this approach was the implementation of a decision tree model using the Chi-Square Automatic Interaction Detection (CHAID) algorithm. In contrast to conventional regression-based approaches, this method enables the derivation of transparent, rule-based decision structures that reflect clinically interpretable combinations of features and support structured prioritization under conditions of diagnostic uncertainty.

The objective of this study was therefore to develop an algorithm based on a CHAID decision tree for the structured risk stratification of Rare Rheumatic Diseases in a Tertiary Referral Setting.

## 2 Methods

### 2.1 Sample

We conducted a retrospective cross-sectional study based on patients referred to the Centre for Rare Diseases (CRD) at Hannover Medical School (MHH) due to an unclear disease and/or suspected rare disease between 10/2019 and 2/2021. All patients were referred to an interdisciplinary case conference (ICC) and to a Model Outpatient Clinic for Rheumatology and Immunology (MRI) at the CRD. Patients were recruited consecutively at the CRD based on predefined inclusion criteria completed diagnostic process, given written consent and complete dataset.

To ensure consistency of score construction, only patients with complete information on all symptom items, laboratory parameters, and medical history variables were included. All analyses were conducted on a complete-case dataset, and no imputation procedures were applied.

All analyses were conducted at the patient level. Each individual was represented by a single observation, regardless of the number of differential or final diagnoses considered during the diagnostic process. In cases where both a rheumatologic diagnosis and other diagnoses were assigned, patients were classified into the RHEUMA group to reflect the presence of a confirmed rheumatologic condition. Diagnostic group composition and frequencies of confirmed diagnoses are reported in the Results chapter.

Highly prevalent rheumatic diseases are usually detected by routine diagnostics and are underrepresented in a highly specialized outpatient clinic. All diagnostic procedures adhered to internationally recognized standards and evidence-based guidelines, including those issued by ACR, EULAR, AWMF, and ASAS.

### 2.2 Ethics

The study was approved by the Ethics Committee of MHH (KFO250, 5,582). The patients gave their written informed consent upon presentation in the Model Outpatient Clinic.

### 2.3 Measures

Demographic characteristics (e.g., age, education level, lifestyle factors), somatic symptoms, medical history, current medications, pre-existing conditions, and comorbidities were recorded using a standardized clinical questionnaire. Data collection was conducted as part of routine clinical assessment at admission by medical specialists and was supplemented with laboratory test results obtained during the diagnostic work-up.

A total of 52 somatic symptoms were assessed in binary format (present/absent). Based on these data, ten symptom-domain sum scores were constructed to capture different aspects of multisystem involvement, each reflecting a clinically defined domain. Symptom grouping was based on organ-system oriented clinical assessment principles. Functional neurological symptoms were categorized in accordance with ICD-11 conceptualizations of functional sensory symptoms (see Supplementary material 1).

Rheumatic pain (RP_SC)
Musculoskeletal symptoms (MS_SC)
Neurological symptoms (NE_SC)
Neuro-functional symptoms (NF_SC)
General symptoms (GE_SC)
Glandular symptoms (GL_SC)
Pulmonary symptoms (PU_SC)
Gastrointestinal symptoms (GA_SC)
Renal symptoms (RE_SC)
Cardiac Symptoms (CA_SC)

The assignment of individual symptoms to the respective clinical scores is provided in Supplementary material 1. To support differential diagnostic stratification in different risk-groups, two higher-order composite scores were constructed to reflect organ- and system-related pathology. The systemic-organ composite score (SO_COM) integrated RE_SC, PU_SC, and GE_SC to capture generalized internal involvement, while the organ-specific composite score (OR_COM) focused on localized pathologies, especially gastrointestinal and pulmonary symptoms (GA_SC, PU_SC). This organ-system based aggregation was guided by the clinical observation that rare inflammatory rheumatic diseases frequently present with systemic and multi-organ involvement rather than isolated organ-specific manifestations. The distinction between systemic-organ and organ-specific composite scores was therefore intended to reflect clinically relevant patterns commonly considered in rheumatologic differential diagnosis. Composite and domain-specific scores were defined *a priori* based on clinical and pathophysiological considerations, with the aim of reflecting typical patterns of organ-system involvement in rheumatologic assessment (2–4, 12, 13, 17–19).

The construction of these scores was not informed by outcome-driven feature selection, model optimization, or post-hoc adjustment. All contributing symptoms were equally weighted to ensure transparency and facilitate interpretability in a clinical context. The predefined score structure was maintained throughout the analysis, and no data-driven modifications or threshold adjustments were performed after inspection of group differences. Group allocation (RHEUMA vs. OTHER) was determined independently through interdisciplinary clinical consensus and was not influenced by the constructed scores.

To ensure clinical feasibility and reduce model complexity, all symptoms were equally weighted, with each contributing one point to the corresponding sum or composite score.

In addition, three laboratory-based composite scores were developed, informed by international classification standards (EULAR/ACR criteria) and existing diagnostic models:

The rheumatologic serology score (RS_Lab) included:

- Rheumatoid factor (RF): positive if >14 IU/mL
- Anti-CCP antibodies: positive if >7 U/mL
- Complement C3: decreased if <0.90 g/L
- Complement C4: decreased if <0.10 g/L
- Anti-alpha-fodrin antibodies: positive if >15 U/mL
  → Each pathological finding contributes 1 point (Range: 0–5).

The biochemical composite score (BC_Lab) reflected potential hepatic and renal pathology and included:

- GPT (ALT): elevated if >50 U/L (men), >35 U/L (women)
- Creatinine: elevated if >1.3 mg/dL (men), >1.1 mg/dL (women)
  → Each pathological finding contributes 1 point (Range: 0–2).

The immunoglobulin profile score (IG_Lab) was composed of:

- IgG: elevated > 16 g/L, IgG4: elevated > 1.4 g/L
- IgM: elevated > 2.30 g/L
  → Each pathological finding contributes 1 point (Range: 0–2).

Symptom-domain scores were initially calculated as raw sum scores reflecting the number of reported symptoms within each domain. For statistical analyses, these scores were subsequently z-standardized to account for differences in the number of contributing items and to facilitate comparability across domains. Standardization was applied solely for statistical modeling purposes; all clinical interpretations refer to relative symptom burden rather than absolute symptom counts. For descriptive group comparisons and regression-based analyses, z-standardized score values were used to account for differences in the number of contributing items and to facilitate comparability across domains. For the CHAID analysis, however, non-standardized (raw) score values were used to preserve the clinical interpretability of the resulting decision rules. Accordingly, all split thresholds shown in the decision tree refer to raw score values rather than standardized metrics. Imaging findings were not included in the decision tree model because imaging procedures were not available in a standardized and complete format across all patients. As imaging was performed selectively according to individual diagnostic pathways rather than systematically as part of a uniform assessment protocol, inclusion of imaging variables would have introduced substantial heterogeneity and missingness. The model was therefore restricted to clinical questionnaire data, medical history variables, and routinely available laboratory parameters.

### 2.4 Statistical methods

Statistical analyses were conducted to describe associations between clinical characteristics (symptom scores, laboratory indices, and medical history variables) and diagnostic group assignment (RHEUMA vs. OTHER). Descriptive statistics are reported as means ± standard deviations for continuous variables and as frequencies and proportions for categorical variables. Group differences were explored using appropriate inferential tests (e.g., chi-square tests, t-tests, or non-parametric equivalents, as applicable).

Given the exploratory nature of the study and the intercorrelated structure of symptom variables, *p*-values from group comparisons are interpreted descriptively and not as confirmatory evidence. No formal adjustment for multiple testing was applied beyond the Bonferroni-adjusted splitting procedures inherent to the CHAID algorithm. Symptom and laboratory indices were expressed as z-scores to account for unequal item numbers. Group comparisons between RHEUMA and OTHER were conducted for descriptive and exploratory purposes.

To model a structured approach for risk stratification, a decision tree was constructed using the Chi-Square Automatic Interaction Detection (CHAID) algorithm. CHAID is a tree-based method that recursively partitions the data based on statistically significant associations between predictors and the outcome, allowing for multi-level splits and the identification of interaction patterns. In the present study, the CHAID model was used as an exploratory tool to identify clinically interpretable combinations of symptom and laboratory features associated with diagnostic group assignment. Model complexity was constrained by limiting tree depth to three levels and by applying predefined stopping criteria, including minimum node sizes and Bonferroni-adjusted significance thresholds. The resulting decision structure was interpreted as a transparent, rule-based framework for organizing clinical information and supporting structured differentiation under diagnostic uncertainty, rather than as a predictive model intended for direct clinical application. In addition, a sensitivity analysis excluding patients with Sjögren’s disease was conducted to assess the stability of the decision tree structure with respect to diagnostic subgroup composition. For the CHAID analysis, non-standardized (raw) score values were used to preserve the clinical interpretability of the resulting decision rules. Full decision rules and node characteristics are reported in Supplementary material 2.

#### 2.4.1 Internal validation and calibration

Internal validation of the CHAID model was performed using ten-fold cross-validation to assess model stability and generalization within the available dataset. The cross-validated misclassification rate (risk estimate) and its standard error were used as primary indicators of model robustness. Apparent performance metrics, including classification accuracy and area under the receiver operating characteristic curve (AUC), are reported descriptively. It should be noted that cross-validation within a single dataset provides only a limited assessment of generalizability and does not replace external validation. Accordingly, performance estimates derived from the present analyses are interpreted as indicative of internal model behavior rather than as evidence of reproducible predictive performance. For the logistic regression model, internal robustness of parameter estimates was assessed using non-parametric bootstrapping with 1,000 resamples, providing bias-corrected confidence intervals for odds ratios. Discrimination performance was evaluated descriptively using receiver operating characteristic (ROC) analysis. Calibration of the CHAID model was assessed descriptively by comparing predicted probabilities within terminal nodes to observed outcome proportions. Given the node-based estimation approach, calibration results are interpreted as descriptive indicators rather than as formal measures of model fit.

## 3 Results

A total of *N* = 332 patients were initially assessed for eligibility. Of these, *N* = 128 patients could not be included in the evaluation for various reasons: (a) incomplete diagnostic process during evaluation period (period ended 4/2023) due to failure to attend follow-up appointment on the part of the patient, rejection of recommendations regarding further diagnostic, long waiting times for specific diagnostics due to the pandemic, lack of patient feedback and completion of documents, (b) lack of written consent. *N* = 27 patients were excluded due to incomplete dataset. Additional *N* = 4 patients were not included in the final analysis due to incomplete diagnostic processes or referral to other treatment centers (see Flow Chart Participants, Supplementary material 3), resulting in a final analytic sample of *N* = 173.

A confirmed rheumatologic diagnosis was established in *N* = 90 patients, whereas *N* = 27 patients received non-rheumatologic diagnoses and *N* = 56 remained without a confirmed diagnosis. The most frequent rheumatologic diagnoses included Sjögren’s disease (*N* = 40) and undifferentiated connective tissue disease (UCTD; *N* = 29) (see Table 1). Among non-rheumatologic diagnoses, neurological disorders represented the largest subgroup (see Supplementary material 4).

**Table 1:** Overview of rheumatological-immunological diagnoses sorted by frequencies.

Diagnosis	*n*
Sjögren’s disease	40
Undifferentiated connective tissue disease (UCTD)	29
Axial Spondyloarthritis	7
Cryoglobulinemia/Cryoglobulinemic vasculitis	5
Psoriatic arthritis	2
Rheumatoid arthritis (RA)	2
Mixed connective tissue disease (MCTD)	1
Vasculitis associated with undifferentiated connective tissue disease	1
Systemic lupus erythematosus (SLE)	1
Chilblain lupus erythematosus	1
Immunodeficiency with autoimmune manifestations	1
Eosinophilic granulomatosis with polyangiitis (EGPA)	1
Polymyositis	1
SAPHO syndrome	1
Systemic sclerosis	1
Sarcoidosis	1

**Figure 1:**
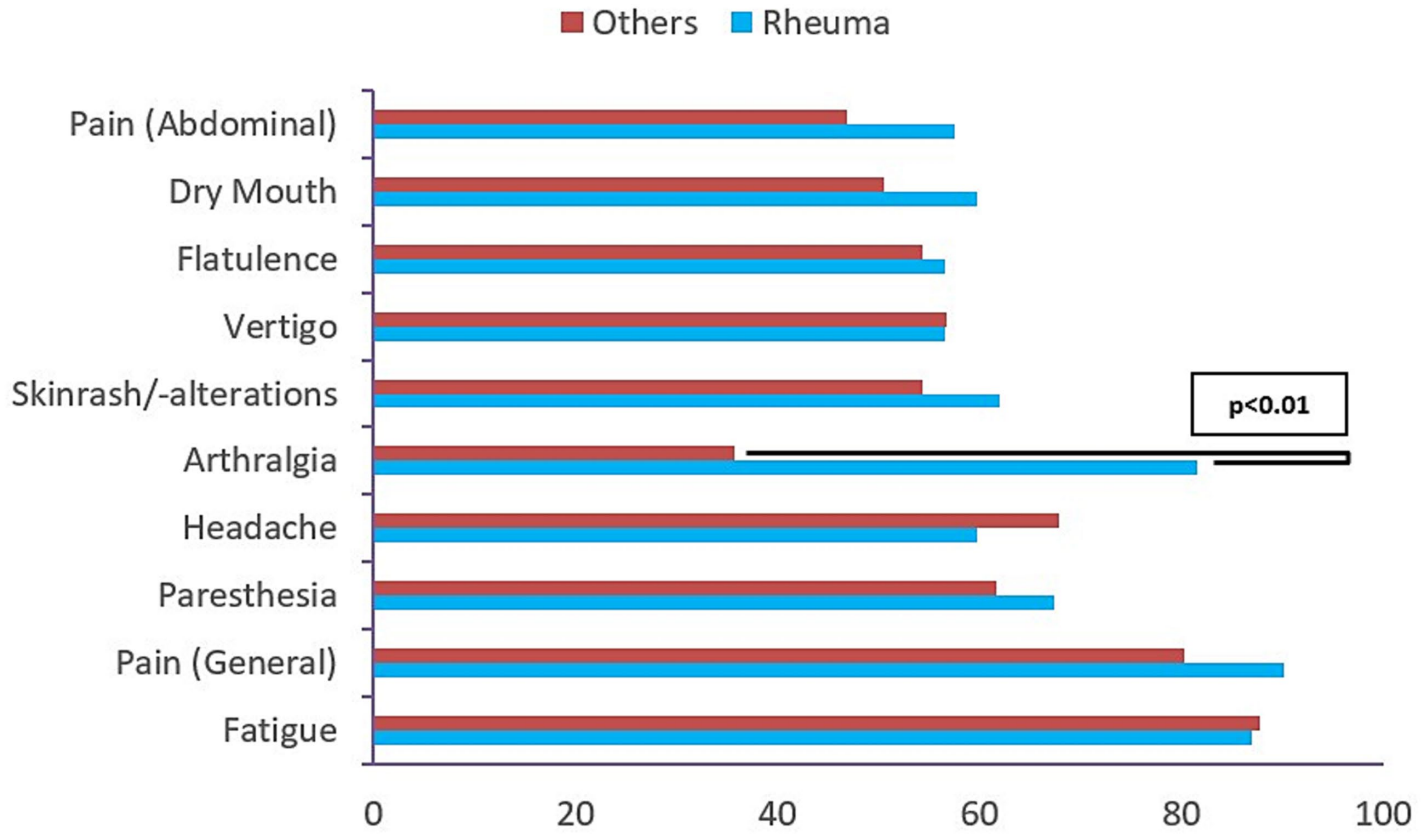
Most common symptoms stratified by diagnostic groups. The figure displays the relative frequency (%) of patient-reported symptoms among individuals with confirmed rheumatologic diagnoses (RHEUMA) and those with other or no final diagnoses (OTHER). Only the most frequently reported symptoms are shown. Bars represent percentages within each diagnostic group.

Both diagnostic groups exhibited complex symptom profiles consistent with multisystem involvement. Patients in the RHEUMA group reported a higher overall number of symptoms compared to the OTHER group (see Table 2). Fatigue and generalized pain were the most frequently reported symptoms in both groups (see Figure 1).

**Table 2:** Demographic and clinical characteristics compared for RHEUMA vs. other.

Characteristics	RHEUMA *N* = 90	Other *N* = 83	Odds ratio (CI)	*p*-value
Demographics				
Age – mean/± std	50.1 ± 14.4	48.4 ± 15.6	1.01 (0.99–1.03)	0.446
Sex – female no. (%)	67 (74.4)	49 (59.0)	2.02 (1.06–3.90)	0.031
Retired no. (%)	28 (31.1)	25 (30.1)	1.05 (0.55–2.00)	0.888
Education – High-school-degree no. (%)	29 (32.2)	24 (28.9)	1.17 (0.61–2.24)	0.637
Migration-History no. (%)	25 (27.8)	18 (21.7)	1.39 (0.69–2.79)	0.354
Smokers – never no. (%)	47 (52.2)	41 (49.4)	0.89 (0.49–1.62)	0.710
Alcohol_consumption – strong no. (%)	5 (5.6)	6 (7.2)	0.76 (0.22–2.57)	0.652
BMI – mean±std	24.8 ± 4.36	24.9 ± 5.67	0.99 (0.94–1.10)	0.835
Rheumatic pain symptoms				
Arthralgia no. (%)	72 (80.0)	32 (38.6)	6.38 (3.23–12.58)	<0.001
Myalgia no. (%)	68 (75.6)	49 (59.0)	2.15 (1.12–4.11)	0.020
Musculosceletal symptoms				
Tendon Pain no (%)	41 (45.6)	22 (26.5)	2.32 (1.22–4.40)	0.009
Morning Stiffness no. (%)	34 (37.8)	23 (27.7)	1.58 (0.83–3.01)	0.159
Muscle Cramps no. (%)	37 (41.1)	32 (38.6)	1.11 (0.61–2.05)	0.732
Number of symptoms				
No. of Symptoms, mean (std.)	19.49 (±8.81)	15.86 (±8.30)	1.05 (1.01–1.09)	0.007
Symptom – scores				
RP_SC, z_score: mean/± std.	0.46 (±0.94)	−0.50 (±0.81)	3.80 (2.43–5.94)	< 0.001
MS_SC,z_score: mean/± std	0.14 (±1.05)	−0.16 (±0.92)	1.36 (1.00–1.85)	0.049
NE_SC, z_score: mean/± std	0.07 (±0.97)	−0.08 (±1.03)	1.16 (0.86–1.57)	0.333
NF_SC, z_score: mean/± std	0.18 (±1.04)	−0.20 (±0.92)	2.27 (1.19–4.31)	0.012
GE_SC, z_score: mean/± std	0.19 (±0.92)	−0.21 (±1.04)	2.32 (1.23–4.39)	0.009
GL_SC, z_score: mean/± std	0.21 (±0.89)	−0.22 (±1.07)	2.59 (1.33–5.06)	0.005
PU_SC, z_score: mean/± std	0.14 (±1.08)	−0.15 (±0.89)	1.34 (0.99–1.82)	0.063
GA_SC, z_score: mean/± std	0.08 (±1.06)	−0.08 (±0.94)	1.18 (0.87–1.59)	0.293
RE_SC, z_score: mean/± std	−0.01 (±0.91)	0.01 (±1.09)	0.98 (0.73–1.32)	0.890
CA_SC, z_score: mean/± std	0.31 (±1.00)	−0.03 (±1.01)	1.07 (0.79–1.44)	0.670
Composite-scores				
OR_COM, z_score: mean/± std	−0.14 (±0.97)	0.15 (±1.02)	0.63 (0.40–1.01)	0.054
SO_COM, z_score: mean/± std	0.14 (±0.96)	−0.16 (±1.03)	1.86 (1.01–3.46)	0.048
Laboratory indices				
RS_Lab z_score: mean/± std	0.46 (±0.94)	−0.50 (±0.81)	1.77 (1.04–3.01)	0.034
BC_Lab, z_score mean/± std.	−0.10 (±0.96)	0.11 (±1.04)	0.78 (0.50–1.25)	0.305
IG_Lab, z_score: mean/± std	0.12 (±1.12)	−0.13 (±0.83)	1.30 (0.95–1.78)	0.103
Pre-existing conditions				
Autoimmune thyroiditis yes (%)	9 (10.0)	10 (12.0)	0.81 (0.31–2.11)	0.667
Any Autoimmune, yes (%)	46 (51.1)	28 (33.7)	2.05 (1.11–3.80)	0.021
Parotitis, yes (%)	11 (12.2)	3 (3.6)	3.71 (1.00–13.82)	0.038
Co-morbidities				
Periodontitis, yes (%)	49 (54.4)	24 (28.8)	2.94 (1.56–5.52)	<0.001
Polyarthritis, yes (%)	17 (18.9)	14 (16.9)	1.15 (0.53–2.50)	0.729
Degenerative spinal disease, yes (%)	27 (30.0)	24 (28.9)	1.05 (0.55–2.03)	0.876
Asthma, yes (%)	26 (28.9)	10 (12.0)	2.97 (1.33–6.62)	0.006
Depression, yes (%)	23 (25.6)	24 (28.9)	0.84 (0.43–1.65)	0.620
Psychiatric, other, yes (%)	10 (11.1)	05 (6.0)	1.95 (0.64–5.96)	0.235

Group comparisons include sociodemographic variables, rheumatic and musculoskeletal symptoms, total number of symptoms, symptom-scores-specific z-scores, laboratory indices z-scores, and pre-existing conditions. Data are presented as means ± standard deviations or absolute numbers with percentages, as appropriate. Odds ratios (OR) with 95% confidence intervals (CI) and *p*-values are reported for between-group comparisons.

With the exception of arthralgia (RHEUMA: 80.0% vs. OTHER: 38.6%, *p* < 0.001), no significant group differences were observed among the most prevalent symptoms, indicating broadly similar symptom patterns across diagnostic categories. The prevalence of any psychiatric disorders was the same in both groups (RHEUMA: 26.7% vs. OTHER: 31.3%, *p* > 0.05). On average, participants reported symptoms across more than six domains (RHEUMA: 6.72 vs. OTHER: 6.14, *p* = 0.023), with slightly greater heterogeneity in the RHEUMA group. Patients in the RHEUMA group showed higher scores in several symptom domains, including rheumatic pain (RP_SC), musculoskeletal (MS_SC), neuro-functional (NF_SC), glandular (GL_SC), and general symptoms (GE_SC) (see Table 2). Notably, 18% of patients in the RHEUMA group presented without RP_SC symptoms, while approximately 40% of the OTHER group reported at least one such symptom. RP_SC scores correlated significantly only with MS_SC (r = 0.28, *p* < 0.001). A significant group difference was also found in pre-existing autoimmune conditions, reported by 51.1% of the RHEUMA group versus 33.7% of the OTHER group (*p* = 0.021). This factor was moderately associated with RP_SC (r = 0.24, *p* = 0.003) and total symptom burden (r = 0.31, *p* < 0.001). Additionally, significantly higher rates of parotitis (12.2% vs. 3.6%, *p* = 0.038) and periodontitis (54.4% vs. 28.8%, *p* < 0.001) were observed in the RHEUMA group.

### 3.1 Laboratory findings

A significant RHEUMA vs. OTHER group difference was observed for the RS_Lab composite score (see Table 2). Several laboratory parameters were frequently observed in both groups, including elevated ANA titers and reduced complement levels; however, these findings did not differ significantly between diagnostic categories. Elevated IgM concentrations >2.30 g/L and ESR > 30 mm/h were observed significantly more frequently in the RHEUMA group (11.1% vs. 2.4%; *p* < 0.05; respectively 5.6% vs. 0%, *p* < 0.05). No further significant differences were identified across the remaining biochemical, rheumatological, or immunoserological markers assessed. Reported rheumatic pain (RP_SC) showed no significant correlation with any of the laboratory scores in either group (correlation coefficients ranging from r = −0.03 to r = 0.04; all *p* > 0.05).

### 3.2 Decision tree model

**Figure 2:**
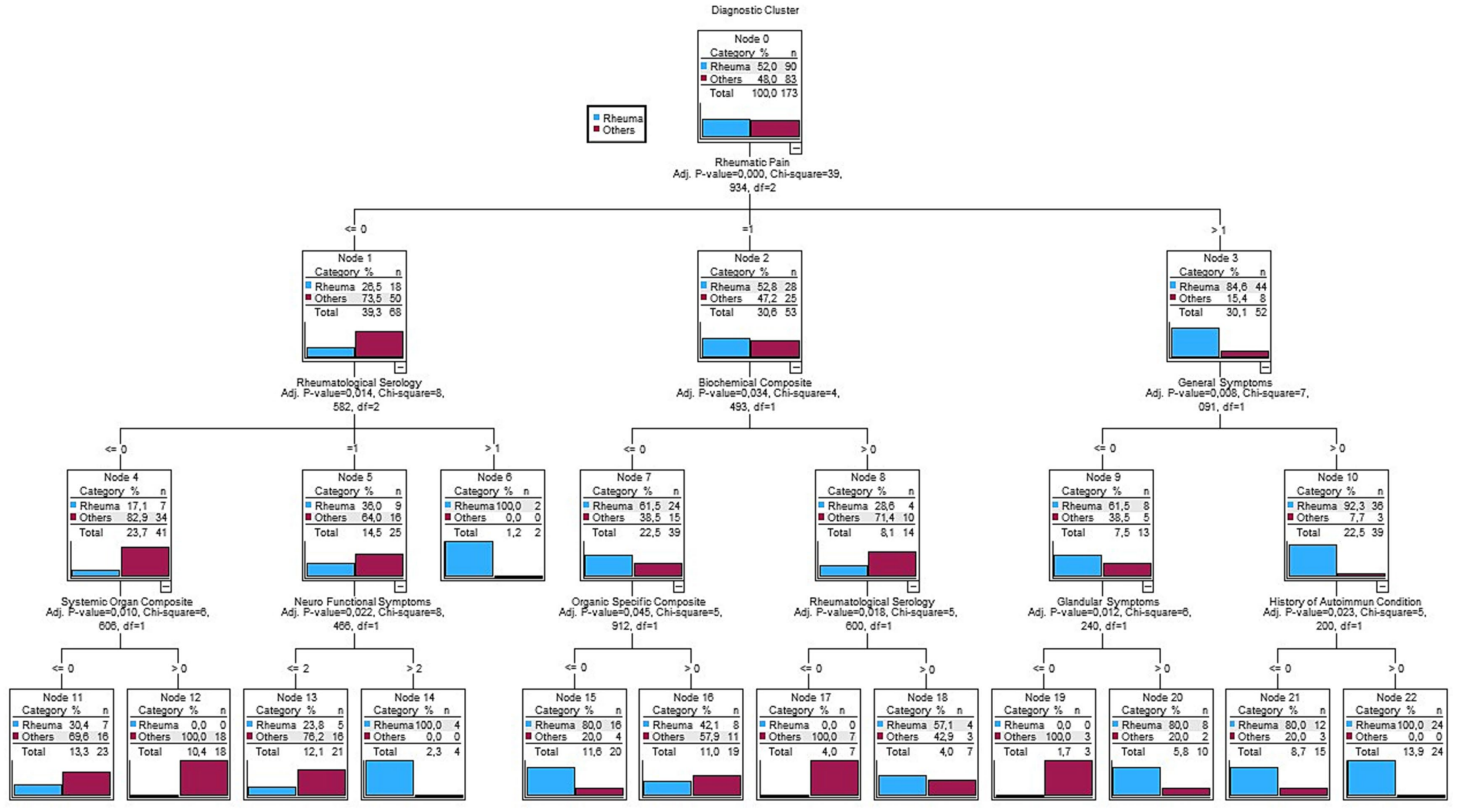
Decision tree for risk stratification of rheumatologic diagnoses based on CHAID analysis. The figure displays the CHAID-based decision tree for differentiation between rheumatologic (RHEUMA) and non-rheumatologic (OTHER) diagnoses. Nodes represent patient subgroups defined by sequential splits based on symptom-domain scores, laboratory indices, and medical history variables. For each node, the total number of patients (n) and the proportion of patients with confirmed rheumatologic diagnoses (%) are shown. Splitting thresholds are based on score values derived from the respective clinical measures (e.g., RP_SC = 0/1/2; RS_Lab = 0/1/>1). All split thresholds shown in the decision tree are based on raw (non-standardized) score values. Abbreviations: RP_SC = Rheumatic Pain Score; GE_SC = General Symptoms Score; NF_SC = Neuro-functional Symptoms Score; RS_Lab = Rheumatologic Serology Score; BC_Lab = Biochemical Composite Score; SO_COM = Systemic Organ Composite; OR_COM = Organ-Specific Composite; HOA_SC = History of autoimmune conditions.

The decision tree model for differentiating between RHEUMA and OTHER is summarized in Figure 2. The CHAID algorithm incorporated selected symptom-domain scores (RP_SC, GE_SC, NF_SC), composite scores (SO_COM, OR_COM), laboratory indices (RS_Lab, BC_Lab), and a medical history variable (history of autoimmune conditions). The resulting model comprised 13 terminal nodes across three hierarchical levels.

The Rheumatic Pain Score (RP_SC) emerged as the primary splitting variable, stratifying the sample into subgroups with different observed proportions of rheumatologic diagnoses. Additional splits were based on general symptoms (GE_SC), neuro-functional symptoms (NF_SC), composite scores, laboratory indices, and autoimmune history, reflecting combinations of clinical features associated with diagnostic group assignment. Terminal nodes were interpreted descriptively in terms of the observed proportion of patients with confirmed rheumatologic diagnoses within each subgroup. Subgroups characterized by higher RP_SC values in combination with additional clinical features showed higher observed proportions of RHEUMA cases, whereas lower RP_SC values combined with alternative patterns were associated with lower observed proportions. The CHAID-based decision tree yielded an apparent classification accuracy of 81.5% (risk estimate = 0.185, SE = 0.030) and an area under the receiver operating characteristic curve (AUC) of 0.893. Ten-fold cross-validation resulted in a misclassification rate of 0.272 (SE = 0.034), corresponding to a cross-validated accuracy of 72.8%, indicating moderate optimism in apparent performance estimates. For comparison, a logistic regression model using the same predictors was estimated (see Section 3.3). While the decision tree showed higher apparent discrimination within the present dataset, both models reflect internal model behavior and should be interpreted descriptively. Overall, the decision tree provides a structured and transparent representation of how combinations of symptom burden, laboratory findings, and medical history variables are associated with diagnostic group assignment within this highly preselected tertiary care cohort. Additional sensitivity analyses excluding the higher-order composite organ scores (SO_COM and OR_COM) are provided in Supplementary material 5. A sensitivity analysis excluding patients with Sjögren’s disease showed that the overall tree structure remained stable, with the Rheumatic Pain Score retained as the primary splitting variable and key second-level splits (rheumatological serology, biochemical composite score, and general symptoms) preserved. Model performance was slightly reduced but remained within a comparable range (accuracy 78.8%, AUC 0.861). A detailed representation of all node-specific decision rules and subgroup characteristics is provided in Supplementary material 2.

### 3.3 ROC curve analysis

**Figure 3:**
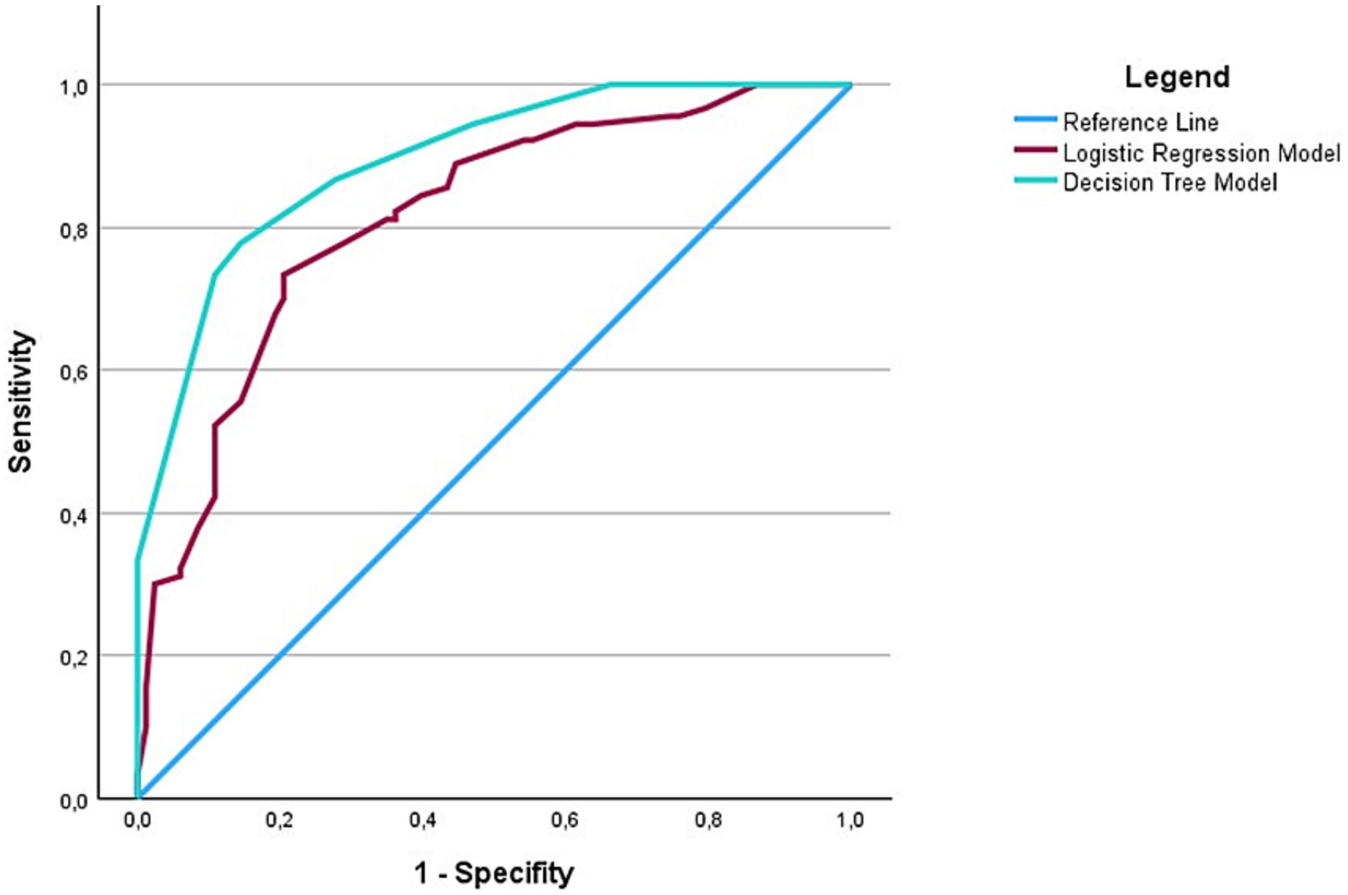
The receiver operating characteristic curves for the decision tree and logistic regression model.

To provide a parametric reference, a binary logistic regression model was estimated using the same set of predictors as included in the CHAID model. In the multivariable model, selected symptom-domain and laboratory variables were associated with diagnostic group assignment. The logistic regression model yielded an apparent classification accuracy of 76.3%, with a sensitivity of 77.8% and a specificity of 74.7%. Apparent discrimination performance was reflected by an area under the receiver operating characteristic curve (AUC) of 0.823 (95% CI: 0.761–0.885). The CHAID model showed a higher apparent AUC (0.893) within the present dataset. However, both estimates reflect apparent model performance and are subject to optimism due to model fitting within a single sample. Accordingly, differences in discrimination between the models are interpreted descriptively and do not provide evidence of superior predictive performance. To assess the stability of regression coefficients, non-parametric bootstrap validation (1,000 resamples) was performed. Bias-corrected confidence intervals remained stable for the primary predictors, indicating internal robustness of the regression model parameters. Figure 3 presents the receiver operating characteristic curves for both models.

## 4 Discussion

In this study, patients referred to a tertiary Center for Rare Diseases with unclear multisystem complaints exhibited a high overall symptom burden with largely similar symptom patterns across diagnostic groups. Individual symptoms and laboratory parameters showed limited discriminative value, whereas aggregated symptom-domain and laboratory scores differed between patients with confirmed rheumatologic diagnoses and patients without confirmed rheumatologic diagnoses or non-rheumatological diagnoses. The CHAID-based decision tree identified combinations of symptom burden, laboratory findings, and medical history variables that were associated with differing proportions of rheumatologic diagnoses. Apparent model performance indicated moderate discrimination, whereas cross-validation suggested reduced accuracy.

The observed overlap of symptom and comorbidity patterns between patients with and without confirmed rheumatologic diagnoses is consistent with previous studies highlighting the diagnostic complexity of inflammatory rheumatic diseases in tertiary care settings. In particular, frequently reported, non-specific symptoms such as fatigue, diffuse pain, and functional complaints (e.g., dizziness or dysphagia), as well as common comorbidities such as depression and broader systemic manifestations associated with chronic inflammatory diseases, are not specific to rheumatologic conditions and contribute to diagnostic uncertainty (20–22). Autoimmune comorbidities are also common in patients with rheumatologic diseases and may further increase diagnostic complexity (23–25). In addition, structural conditions such as degenerative spinal diseases and generalized osteoarthritis may present with overlapping symptom profiles, further complicating clinical differentiation. This reflects the often non-specific and systemic nature of symptom presentations, which frequently extend beyond classical organ boundaries and complicate differentiation from non-rheumatologic conditions (5, 6, 15, 26–30). In this context, the decision tree may provide a structured and transparent representation of clinically relevant feature combinations. Its potential value therefore lies primarily in the transparent organization of complex clinical information rather than in predictive performance.

Against this background, the limited differentiation achieved by individual symptoms and laboratory parameters underscores the importance of integrative approaches that consider combinations of clinical features rather than single indicators. Similar observations have been reported in studies of complex autoimmune and rare diseases, where diagnostic classification often depends on patterns of co-occurring symptoms and contextual clinical information rather than on specific markers alone (2, 31–33).

Within this context, the present approach of aggregating symptoms into clinically informed domains and combining them with selected laboratory indices provides a structured way to capture multisystem involvement. Rather than identifying isolated predictors, the decision tree model reflects how combinations of features relate to diagnostic group assignment within this specific clinical setting. This is consistent with broader developments in data-driven approaches to complex diseases, which emphasize pattern recognition and integrative modeling over single-variable associations (31–38).

Importantly, the potential relevance of the decision tree may lie less in predictive performance itself than in its transparent and interpretable structure. In contrast to less interpretable modeling approaches, such as ensemble-based machine learning methods, the rule-based structure of the CHAID model enables explicit representation of how clinical features interact within the stratification process. This structure may support the transparent organization of clinical information in diagnostically challenging cases, particularly in settings characterized by high symptom heterogeneity and diagnostic uncertainty (39, 40). Sensitivity analyses excluding patients with Sjögren’s disease indicated that the overall structure of the decision tree was largely preserved, suggesting that the model is not solely driven by this diagnostic subgroup.

This study has several strengths. The use of a structured, rule-based modeling approach allowed for transparent representation of clinically relevant feature combinations and facilitated interpretability of the resulting decision tree. In addition, the integration of symptom-domain scores, laboratory indices, and medical history variables reflects routine clinical information available in early diagnostic stages.

Several limitations should be considered. The monocentric design and preselected tertiary care cohort limit the generalizability of the findings. In addition, the retrospective design may have introduced selection and information bias. Imaging findings were not included in the decision tree due to their non-standardized, indication-driven use in this cohort. Future studies should assess whether systematically collected imaging data improve model performance. The use of composite scores may introduce abstraction and reduce direct clinical interpretability. However, sensitivity analyses excluding selected composite variables indicated a largely stable decision tree structure. Finally, the model was developed in a cohort with a relatively high proportion of patients with Sjögren’s disease, which may have influenced specific decision pathways and limit transferability to other clinical settings.

Future research should focus on external validation of the proposed decision tree in independent cohorts and across different clinical settings. In particular, validation in both comparable tertiary care populations and less selected patient groups is essential to assess generalizability. Prospective studies are warranted to evaluate the clinical utility of the model and its potential role in supporting structured diagnostic decision-making. In addition, future work should examine whether the integration of further data sources, such as systematically collected imaging findings, improves model performance and interpretability.

## Data Availability

The data used in this study has been pseudonymized, they are not publicly accessible. The raw data supporting the conclusions of this article will be made available by the corresponding author, without undue reservation, to any qualified researcher upon reasoned request.
