# Supplementary material for: A decision tree-based algorithm for structured risk stratification of rare rheumatic diseases in a tertiary referral setting
Source: Front Med (Lausanne). 2026 Jul 2;13:1734483. doi: 10.3389/fmed.2026.1734483 (PMC13372701; doi:10.3389/fmed.2026.1734483)
Supplement: Supplementary file 1 [file Data_Sheet_1.pdf]

**Supplement 1: Symptom Assignment of the Clinical Scores**

| **Clinical Scores** | **Abbreviation** | **Symptoms** |
|---|---|---|
| Rheumatic pain | RP_SC | See table 2 |
| Musculosceletal symptoms | MS_SC | See table 2 |
| Neurological symptoms | NE_SC | Paresthesia, dizziness, trigeminal complaints, paresis/muscle weakness, limitations/walking, gait instability, myoclonus, facial nerve palsy, migraine, anosmia, diplopia, hypacusis |
| Neuro-functional symptoms* | NF_SC | Globus sensation, dysphagia, tinnitus |
| General symptoms | GE-SC | Tendency to infection, fever, edema, bruxism, bleeding tendency, weight loss, weight gain |
| Glandular symptoms | GL_SC | Xerostomia, xerophthalmia, gritty feeling |
| Pulmonary symptoms | PU_SC | Dyspnea, cough/dry, cough/productive |
| Gastrointestinal symptoms | GA_SC | Food intolerance, abdominal pain, bloating, diarrhea, constipation |
| Renal symptoms | RE_SC | Urine/foaming, urine/discolored, urinary retention, incontinence |
| Cardiac symptoms | CA_SC | Palpitations, thrombosis |

* The functional symptoms were assigned empirically using a PCA analysis based on high intercorrelations and in accordance with the ICD_11 classification 6C20.1 Functional sensory symptoms.
