# Supplementary material for: A decision tree-based algorithm for structured risk stratification of rare rheumatic diseases in a tertiary referral setting
Source: Front Med (Lausanne). 2026 Jul 2;13:1734483. doi: 10.3389/fmed.2026.1734483 (PMC13372701; doi:10.3389/fmed.2026.1734483)
Supplement: Supplementary file 2 [file Data_Sheet_2.pdf]

Supplement 2: Final CHAID-Decision-Rules

Software: SPSS, No. 31.0, Outcome Variable: Confirmed rheumatological diagnosis (RHEUMA vs. OTHER); Max. tree depth: 3; Minimum parent node size: 5; Minimum child node size: 2; Splitting criterion: Chi-square test; Significance level: 0.05; Multiple testing correction: Bonferoni adjustment; Handling of missing values: no missing values in predictors; Cross-validation: Ten-fold cross-validation (risk estimate).

Predictor	Abbr.	Definition
Rheumatic Pain Score	**RP_SC**	Sum score of rheumatic pain items (range: [0–2]); categorized as asymptomatic=0/ incomplete=1/ complete=2 based on CHAID-derived cut-offs
General Symptoms Score	**GE_SC**	Sum score of general systemic symptoms (range: 0–7)
Neuro-functional Symptoms	**NF_SC**	Sum score of functional neurological symptoms (range: 0–3)
Rheumatologic Serology	**RS_Lab**	Definition see Measures (range: 0-5)
Biochemical Composite	**BC_Lab**	Definition see Measures (range: 0-2)
Glandular Symptoms	**GL_SC**	Sum score of glandular symptoms (range: 0-3)
Systemic Organ Composite	**SO_COM**	Definition see Measures (range:0-14)
Organ Specific Composite	**OR_COM**	Definition see Measures (range:0-8)
History of autoimmune disease	**HOA_SC**	Any documented autoimmune condition, yes/no (range:0-1)

Final CHAID-Decision Rules

Terminal Node	Decision Rule all conditions must be met	N	Rheuma N (%)	Predicted Probability
Node 1	RP_SC=0	68	18 (26.5)	0.265
Node 2	RP_SC=1	53	28 (52.8)	0.528
Node 3	RP_SC=2	52	44 (84.6)	0.846
Node 4	RP_SC=0 AND RS_Lab=0	41	7 (17.1)	0.171
Node 5	RP_SC=0 AND RS_Lab=1	25	9 (36.0)	0.360
Node 6	RP_SC=0 AND RS_Lab>1	2	2 (100)	1.000
Node 7	RP_SC=1 AND BC_Lab=0	39	24 (61.5)	0.615
Node 8	RP_SC=1 AND BC_Lab>0	14	4(28.6)	0.286
Node 9	RP_SC =2 AND GE_SC=0	13	8 (61.5)	0.615
Node 10	RP_SC=2 AND GE_SC>0	39	36 (92.3)	0.923
Node 11	RP_SC =0 AND RS_Lab=0 AND SO_COM=0	23	7 (30.4)	0.304
Node 12	RP_SC= 0 AND RS_Lab=0 AND SO_COM>0	18	0 (0.0)	0.000
Node 13	RP_SC= 0 AND RS_Lab=1 AND NF_SC<=2	21	5 (23.8)	0.238
Node 14	RP_SC= 0 AND RS_Lab=1 AND NF_SC>2	4	4 (100.0)	1.000
Node 15	RP_SC=1 AND BC_Lab=0 AND OR_Com=0	20	16 (80.0)	0.800
Node 16	RP_SC=1 AND BC_Lab=0 AND OR_COM > 0	19	8 (42.1)	0.421
Node 17	RP_SC=1 AND BC_Lab>0 AND RS_Lab=0	7	0 (0.0)	0.000
Node 18	RP_SC=1 AND BC_Lab>0 AND RS_Lab>0	7	4 (57.1)	0.571
Node 19	RP_SC=2 AND GE_SC=0 AND GL_SC=0	3	0 (0.0)	0.000
Node 20	RP_SC=2 AND GE_SC=0 AND GL_SC>0	10	8 (80.0)	0.800
Node 21	RP_SC=2 AND GE_SC>0 AND HOA_SC=0	15	12 (80.0)	0.800
Node 22	RP_SC=2 AND GE_SC>0 AND HOA_SC=1	24	24 (100.0)	1.000

Risk group classification (defined by first-level split (RP_SC=0/1/2)

**Risk group**	**Terminal nodes**	**RHEUMA probability range**
Low risk	Nodes [11-14]	approx. [0.00]–[100.0]%
Intermediate risk	Nodes [15-18]	approx. [0.00]–[80.0]%
High risk	Nodes [19-22]	approx. [0.00]–[100]%

Risk groups were defined by the first-level split of the CHAID decision tree based on the Rheumatic Pain Score (RP_SC); all terminal nodes descending from each first-level node were assigned to the corresponding low-, intermediate-, or high-risk group, and probability ranges reflect the minimum and maximum node-specific predicted probabilities within each group.
