# Supplementary material for: A decision tree-based algorithm for structured risk stratification of rare rheumatic diseases in a tertiary referral setting
Source: Front Med (Lausanne). 2026 Jul 2;13:1734483. doi: 10.3389/fmed.2026.1734483 (PMC13372701; doi:10.3389/fmed.2026.1734483)
Supplement: Supplementary file 3 [file Data_Sheet_3.pdf]

Supplement 3: Flow Chart Participants

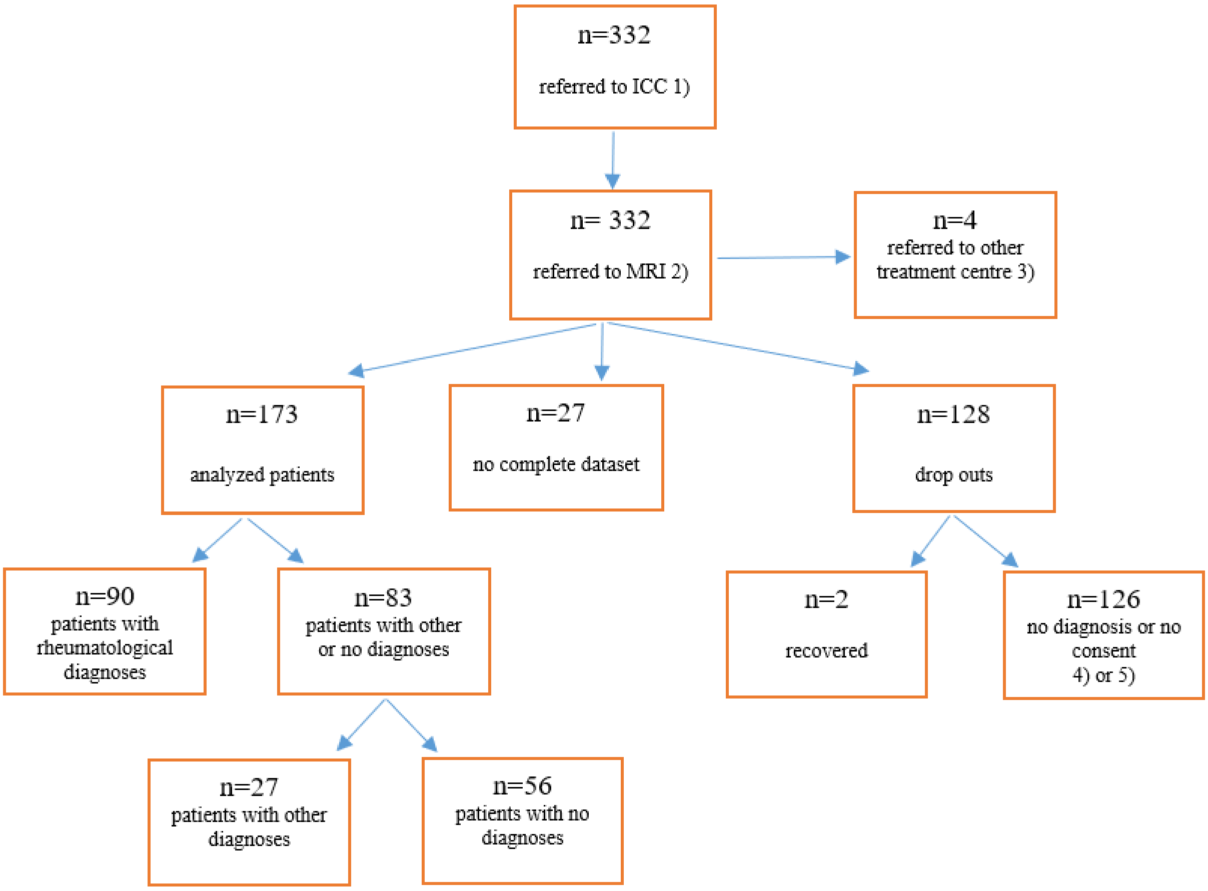

Legend Flow Chart

1) ICC: Interdisziplinary case conference.

2) MRI: Model Outpatient Clinic for Rheumatology and Immunology at the CRD.

3) Three patients referred to other treatment center for organisational reasons, one patient due to extern diagnosis of cancer.

4) Incomplete diagnostic process during evaluation period (period ended 4/2023) due to

- failure to attend follow-up appointment on the part of the patient
- rejection of recommendations regarding further diagnostics
- long waiting times for specific diagnostics due to the pandemic
- lack of patient feedback and completion of documents.

5) Lack of written consent.
