# Supplementary material for: A decision tree-based algorithm for structured risk stratification of rare rheumatic diseases in a tertiary referral setting
Source: Front Med (Lausanne). 2026 Jul 2;13:1734483. doi: 10.3389/fmed.2026.1734483 (PMC13372701; doi:10.3389/fmed.2026.1734483)
Supplement: Supplementary file 4 [file Data_Sheet_4.pdf]

Supplement 4: Overview of Non-Rheumatological Diagnoses

**Diagnosis**
Small fiber neuropathy
CIDP
Multiple sclerosis
Myotonia congenita type Thomsen
Myelitis DD MS with relapsing course
Periodic paralysis with mutation in the SCN4A gene
Suspicion of neurosarcoidosis
FTD
Suspicion of multisystem atrophy, Shy-Drager syndrome
Neuromyotonia, CASPR2-positive
Neurofibromatosis, genetically confirmed
Fatigue syndrome
Metabolic myopathy, MCAD deficiency
Myopathy with elevated IgG4
Primary hyperparathyroidism in parathyroid adenoma
Hypophosphatasia
Alpha-1 antitrypsin deficiency
Chronic spontaneous urticaria
Histamine intolerance

Erythromelalgia
Postural orthostatic tachycardia syndrome
Chronic venous insufficiency
Phenylketonuria, secondary immunodeficiency with IgG deficiency, known intestinal lymphangiectasia with intestinal protein loss syndrome
Type II diabetes mellitus, art. Hypertension
Suspicion of mild cholestatic cholangitis, detection of Enterococcus faecium and Enterococcus casseliflavus in bile
Suspicion of premenstrual syndrome
