# Supplementary material for: A decision tree-based algorithm for structured risk stratification of rare rheumatic diseases in a tertiary referral setting
Source: Front Med (Lausanne). 2026 Jul 2;13:1734483. doi: 10.3389/fmed.2026.1734483 (PMC13372701; doi:10.3389/fmed.2026.1734483)
Supplement: Supplementary file 5 [file Data_Sheet_5.pdf]

Supplement 5: Supplementary Methods and Results: Sensitivity Analysis Excluding Composite Organ Scores (SO_COM and OR_COM)

Rationale

To address potential concerns regarding information leakage arising from the construction and use of higher-order composite scores (SO_COM and OR_COM), we conducted a predefined sensitivity analysis in which these composite variables were excluded from the predictor set. This analysis was designed to assess whether the diagnostic performance and decision structure of the CHAID-based classification model were materially dependent on the inclusion of these composites.

Study Population and Outcome

The sensitivity analysis was performed on the same analytic cohort as the primary analysis, comprising **N = [173] patients**, of whom **N = [90]** were classified as RHEUMA and **N = [83]** as OTHER. The binary outcome variable (confirmed rheumatologic diagnosis vs. other or no diagnosis) was defined at the patient level and was identical to that used in the main model.

Predictor Sets

Two CHAID models were compared:

1. **Primary model (reference model):**
   Included symptom-domain scores, laboratory-based composite scores, medical history variables, and the composite organ scores SO_COM and OR_COM.
2. **Sensitivity model (reduced model):**
   Included the identical set of predictors **except for SO_COM and OR_COM**, which were fully excluded from model training and evaluation.

All remaining predictors (e.g., RP_SC, GE_SC, NF_SC, RS_Lab, BC_Lab, and history of autoimmune conditions) were defined and operationalized identically to the primary analysis.

Model Specification

For both models, identical CHAID settings were applied to ensure comparability: Maximum tree depth: **[3];** Splitting criterion: Chi-square test with Bonferroni-adjusted significance levels. Given the modest sample size and the multi-category nature of CHAID splits, minimum parent/child node sizes were kept at 5/2 to allow clinically meaningful tree growth while preserving model comparability across the primary and sensitivity analyses. We acknowledge that small node sizes may increase variance of cut-points and therefore emphasize cross-validated misclassification rates for internal validation. Minimum parent node size: **[5];** Minimum child node size: **[2];** Handling of missing values: **[no missing values]**

No additional tuning or post-hoc optimization was performed for the sensitivity model.

Performance Evaluation

Model performance was evaluated using the same metrics as in the primary analysis, including: Overall classification accuracy; Sensitivity and specificity, Area under the receiver operating characteristic curve (AUC); Ten-fold cross-validated misclassification rate.

Performance estimates were derived using the full dataset and cross-validation procedures identical to those applied in the primary model.

Supplementary Results

Model Performance

Reported accuracy values refer to apparent (re-substitution) performance, whereas misclassification rates are derived from ten-fold cross-validation. Cross-validation outputs provided by SPSS decision trees were used to report the cross-validated risk estimate (misclassification rate); other metrics (accuracy, sensitivity, specificity, AUC) refer to apparent performance in the full dataset.

Exclusion of the composite organ scores SO_COM and OR_COM resulted in the following performance characteristics: Accuracy: **[79.8%]** (primary model: **[81.5%]**); Sensitivity: **[86.7%]** (primary model: **[77.8%]**); Specificity: **[72.3%]** (primary model: **[85.5%]**); AUC: **[0.880]** (primary model: **[0.893]**); Ten-fold cross-validated misclassification rate: **[28.9%]** (primary model: **[26.3%]**).

Overall, performance metrics differed only marginally between the two models (ΔAUC = 0.013; Δaccuracy = 1.7 percentage points; ΔCV misclassification = 2.6 percentage points), indicating overall robustness with respect to the exclusion of SO_COM and OR_COM. The observed trade-off between sensitivity and specificity suggests a slightly more inclusive classification in the reduced model, while overall discrimination remained stable.

Decision Tree Structure

The overall structure of the CHAID decision tree remained largely unchanged in the sensitivity model. In particular: The primary splitting variable at the root node remained **[RP_SC]**. Key downstream splits involving **[GE_SC / NF_SC / RS_Lab / history of autoimmune conditions – as applicable]** were preserved. The number of terminal nodes was **[12]** in the sensitivity model compared with **[13]** in the primary model. Risk stratification into low-, intermediate-, and high-risk groups showed comparable distributions and clinically interpretable decision pathways across both models.

Conclusion

This sensitivity analysis demonstrates that exclusion of SO_COM and OR_COM does not materially affect the predictive accuracy, risk stratification, or interpretability of the CHAID-based decision model. The robustness of the results supports the validity of the primary findings and mitigates concerns regarding optimistic bias due to composite feature construction. Given the modest sample size, emphasis was placed on cross validated misclassification rates for internal validation.
